# Ultrafine Graphite Scrap and Carbon Blocks Prepared by High-Solid-Loading Bead Milling and Conventional Ball Milling: A Comparative Assessment

**DOI:** 10.1021/acsomega.3c06164

**Published:** 2023-12-08

**Authors:** Chonradee Amnatsin, Waroot Kanlayakan, Siraprapa Lhosupasirirat, Nattarut Verojpipath, Boonsueb Pragobjinda, Tanakorn Osotchan, Chakrit Sirisinha, Toemsak Srikhirin

**Affiliations:** † School of Materials Science and Innovation, Faculty of Science, Mahidol University, Nakhon Pathom 73170, Thailand; ‡ Department of Physics, Faculty of Science, Mahidol University, 272 Rama VI Road, Ratchathewi District, Bangkok 10400, Thailand; § Research Network of NANOTEC at Mahidol University, National Nanotechnology Center, Bangkok 10400, Thailand; ∥ Rubber Technology Research Centre, Faculty of Science, Mahidol University, Nakhon Pathom 73170, Thailand; ⊥ Thai Carbon & Graphite Co., Ltd., 15/2 Phutthamonthon Sai IV Road, Kratumban District, Samutsakorn 74130, Thailand

## Abstract

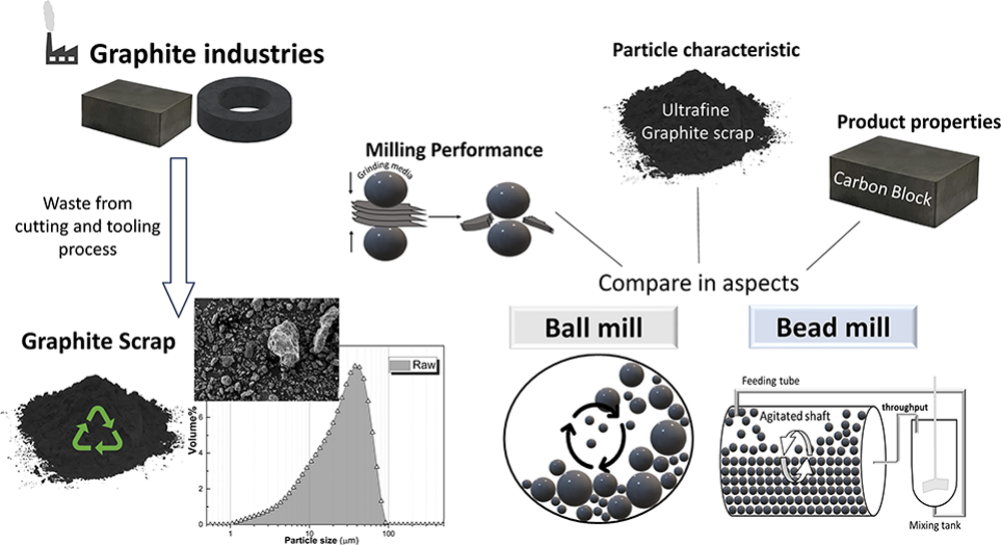

A comparison between the physical characteristics of graphite ultrafine particles and the properties of graphite blocks prepared from graphite scrap using bead and conventional ball milling techniques is presented. Industrial-scale bead milling was used to prepare graphite scrap with an initial particle size *d*_50_ of 24 μm in the ultrafine range of <10 μm. Bead milling can significantly reduce the production time of ultrafine graphite from graphite scrap from 72 h by ball milling to 10 min. Ultrafine graphite scrap prepared from both ball milling and bead milling yields particles with a similar morphology, with a minor difference in crystalline size *L*_a_ and stacking height *L*_c_ observed. Carbon blocks were fabricated from both techniques, yielding carbon blocks with an almost identical microstructure and block density. Blocks from bead milling have slightly higher flexural strength as well as comparable hardness and resistivity. The block’s flexural strength, hardness, and resistivity were 68.37 MPa, 99, and 36.9 μΩ·m, respectively, in a bead-milled carbon block and 61.86 MPa, 95.5, and 38.6 μΩ·m, respectively, for a ball-milled carbon block. Bead milling can be applied for the preparation of ultrafine graphite particles and graphite blocks with production that is 9 times faster for the same ultrafine graphite particle output and final product quality.

## Introduction

Graphite is a layered carbon material in which carbon atoms are arranged in a hexagonal pattern with unique thermal conductivity, electrical conductivity, and lubrication properties. Due to these properties, graphite has been used in a variety of applications, including refractories, friction materials, lubricants, battery anodes, expanded graphite, graphite foil, and nuclear power plants.^1,2^ Graphite is also the dominant material for anodes in the lithium-ion battery industry.^3,4^ In 2022, graphite consumption was reported to be over 72,000 Mts in the United States alone. The amount of recoverable graphite worldwide was estimated at more than 800 million tons.^5^ This graphite scrap was either disposed of or sold as low-grade graphite.

Many research studies used recovered graphite and synthetic graphite to replace natural graphite to recycle graphite waste rather than dispose of it. Graphite was reported to be upcycled in new applications such as polymer composites,^6,7^ nuclear graphite,^8,9^ and bulk graphite.^10,11^ Although most work focused on recycling a recovered graphite anode into an anode for lithium-ion batteries,^12−14^ graphite is consumed largely in battery, refractory, and machinery part applications.^5^ In machinery parts and refractory product fabrication, carbon blocks must initially be formed by a combination of a filler (graphite) and a binder (coal/tar pitch) mixed in the hot kneading process at a constant temperature and then pulverized into powder. Subsequently, the mixture is either shaped by cold compression, hot compression, or extrusion to a green body. The green body block is carbonized by the heat treatment process.^15−17^

According to the ASTM D8075-16 standard,^18^ carbon blocks have been categorized by the size range of the filler particle (graphite). From coarse to microfine, blocks from smaller fillers (ultrafine and microfine) have been used in high-performance applications due to their superior mechanical properties.^10,19−21^ Reducing the particle of graphite scrap into a smaller class as the ultrafine range can increase the value of graphite scrap and make it available for a wider range of applications. Recycling graphite scrap from the manufacturing process will lead to a green industry and zero-waste production.

There are several particle size reduction techniques available such as rod mills, rotary mills, ball mills, stirred media mills, etc.^22−24^ The comminution technique that has been used commercially is ball milling. Ball milling is a process that works by rotating the grinding media (often aluminum or zirconium balls) in a grinding chamber to grind the particles inside. The process reduces particle size by the shearing force and the gravitational force of grinding media falling from the top of the chamber to the bottom. However, a limitation of ball milling is the long milling time needed. The process could take several days to crush coarse particles into small particles.^25,26^ To complement these disadvantages in ball milling, a high-efficiency milling process was developed. Due to the advantages of bead milling, the idea of switching from a prolonged milling process such as ball milling to a high-efficiency process such as bead milling has caught the attention of researchers.

Bead milling, the so-called stirred media mill, is a technique that was developed based on the working principle of ball milling. However, bead milling uses smaller grinding media ranging from 3 mm to micrometer size and higher milling speed (up to thousands of rpm). Because of the difference in grinding media size and milling speed, bead milling requires a shorter production time than ball milling due to the higher energy used in bead milling.^27−30^ Based on other studies concerning the energy efficiency between the ball and stirred media mill^31,32^ due to the shorter milling time required in a bead mill, it was found that a stirred media mill was more energy efficient than a ball mill; replacing ball milling with bead milling could shorten the production time and lower the energy consumption in production by approximately 30–40%.^33^ The technique is also available in various sizes of commercial production.

Ball milling has been used extensively in research involving graphite deagglomeration, delamination, and size reduction.^34−36^ Studies have also used bead milling for graphite delamination to produce graphene^37,38^ and graphite particle size reduction.^39^ However, the bead milling process should be able to utilize graphite scrap at high-concentration milling to be a comparable process to ball milling on an industrial scale, although information concerning the use of bead milling in high-graphite-concentration milling on a large production remains limited.

Bead milling was proposed as a new particle reduction process replacing ball milling to reduce the production time. Bead milling was used to reduce the size of graphite scrap with a *d*_50_ of 24 μm to the ultrafine range (<10 μm). Parameters such as process time and slurry viscosity were compared with ball milling to evaluate the performance of bead milling. Output particle characteristics, such as morphology and crystallinity, were compared to observe the differences. Finally, the milled ultrafine graphite from both processes was fabricated into carbon blocks and compared in terms of block microstructure and electrical and mechanical properties (density, flexural strength, and hardness) to verify whether changing the particle reduction process to bead milling had an impact on the final application as a carbon block.

## Experimental Section

### Raw Materials and Chemicals

Graphite scrap was collected from the shaping and tooling stage in the production line (Thai Carbon and Graphite Co., Ltd., Thailand) of an isotropic graphite block as a byproduct. Graphite scrap was sieved with a #60 mesh before being used in this experiment. The average particle size of the raw graphite scrap (*d*_50_) was 24 μm.

Sodium dodecyl sulfate (Sigma-Aldrich, CAS no. 151-21-3) and lignosulfonate (received from Thai Carbon and Graphite Co., Ltd.) were used in this work as surfactants for the preparation of graphite scrap slurry for both ball milling and pilot-scale bead milling.

### Milling

#### Ball Milling

The ball milling process was performed with a 1 × 1 m cylinder milling chamber operated using 4 sizes of zirconia balls (10, 15, 20, and 25 mm) with a ball ratio of 1:1 (400 kg in total). Graphite scrap slurry was prepared with 200 kg of water and graphite scrap powder (150 kg), along with 0.5% SDS and 1.5% LS (% relative to graphite weight). Following 1 h of premixing, milling at a speed of 25 rpm was commenced. At the 30 and 50 h marks, water was added to the grinding process to reduce viscosity; a total of 30 kg was added to the system. The final solid loading in ball milling was 40 wt %.

#### Bead Milling

Commercial-scale bead milling (DISCUS 20, Netzsch, German) employing 0.8 mm zirconia bead grinding media (filled 80 vol % of the agitated chamber) at 1,500 rpm was used to utilize the graphite scrap slurry. For the slurry, the solid loading of graphite scrap slurry used in the pilot-scale bead milling process was 23 wt % graphite scrap powder (30 kg) with 100 kg of water and 0.5% SDS and 2% LS. The graphite scrap slurry mixture was premixed for 1 h before undergoing bead milling at 1,500 rpm. It should be noted that the machine was used only at one-third of its maximum capacity during this operation.

The comminution processes were carried out until both techniques produced particle sizes that were smaller than 10 μm in *d*_50_. Slurry samples were collected from the throughput of the bead milling process and inside the milling chamber of the ball milling.

### Block Fabrication

Ultrafine graphite scrap produced from ball milling and industrial-scale bead milling was dried and pulverized by a pin mill for block fabrication. To fabricate carbon blocks, 29 wt % ultrafine graphite scrap was first mixed with 41 wt % coal tar pitch and 30 wt % amorphous carbon in hot kneading at 180 °C. The mixture was pulverized and shaped into carbon blocks by compression molding at 1,000 psi to the block with a dimension of 40 × 80 × 100 cm^3^. The green block was then carbonized by heat treatment at 950 °C. After the heat treatment, carbon blocks were cut into small specimens of 1 × 1 × 10 cm^3^ for further property characterization, including microstructure, density, flexural strength, hardness, and resistivity.^40^

### Slurry and Particle Characterizations

#### Particle Size

Collected graphite scrap slurry samples from the ball milling and bead milling processes were used for particle size characterization. The particle size was measured by using a laser diffraction particle size analyzer (Mastersizer 3000E, Malvern). From this technique, size distribution and volume-based size parameters (*d*_10_, *d*_50_, and *d*_90_) were collected.

#### Slurry Viscosity

The viscosity of the slurry was measured by a viscometer (Brookfield, DVE) with spindle no. LV-S3 at 50 rpm. The measurement was repeated 3 times and averaged.

#### Particle Morphology and Block’s Microstructure

The particle morphology and block microstructure were characterized by a scanning electron microscope (JSM-IT500, Jeol) with an accelerating voltage of 10 kV. Graphite scrap particles were redispersed in isopropanol with a 0.01 wt % concentration; 10 μL of the graphite dispersion was dropped on a Si wafer and heated at 120 °C for 4 days. For the carbon block, block specimens were cut into 1 × 1 × 1 cm^3^ and then polished with no. 1200 sandpaper on the surface. The polished surface and cross section of the carbon block were observed.

#### Particle Crystallinity

The crystallinity of the particles was characterized by X-ray diffraction (D8 Discover, Bruker, Germany) using Cu Kα radiation at λ 1.5406 nm running conditions followed by a 0.01° step side in a 2θ scanning range of 10–90°. The crystalline parameters were calculated following the equation below,^41−45^ where β represents the full width at half-maximum (fwhm) of the diffraction peak and *k* is constant with *k*_1_ and *k*_2_ equal to 1.84 and 0.94, respectively.

Bragg’s law for the interlayer spacing (*d*_002_)
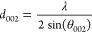
1

The Scherrer equation for the crystalline size (*L*_a_)


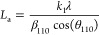
2

For the stacking height (*L*_c_)
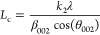
3

### Mechanical Properties of the Carbon Block

The density of the carbon block was calculated by using the weight and volume of the final carbon block. Afterward, the hardness of the carbon block was measured by a SATO hardness tester (SK SATO, Japan) in the shore d mode following ASTM C886-21.^46^ Electrical resistivity was measured by a four-point probe test following ASTM C661-21.^47^ Lastly, the flexural strength was measured by three-point bending (Ametek EZ50, Lloyd Instruments, United Kingdom) following ASTM D7972-14.^48^ For each test, the carbon block was cut into 6 smaller specimens with dimensions of 1 × 1 × 10 cm^3^ before measurement.

## Results and Discussion

Due to the coarse particle size of raw graphite scrap particles (*d*_10_ of 5.91 μm, *d*_50_ of 23.6 μm, and *d*_90_ of 49.6 μm), the size reduction technique was required to produce ultrafine graphite scrap particles for higher-quality carbon block products. Different industrial size reduction techniques have been utilized for ultrafine graphite scrap production, such as the ball milling process and the novel bead milling technique. Moreover, comparisons in terms of milling performance, particle characteristics, and carbon block properties of each technique were investigated. Particle reduction processes were performed using the optimal milling conditions and slurry formulation.

A surfactant helps control viscosity build-up during the milling process due to an increase in the total surface area of the particle by preventing milled graphite particles from reagglomeration. Our previous publication^40^ emphasized the effect of surfactants (SDS, LS, and LS-SDS) on the ball milling performance for the preparation of ultrafine graphite scrap production from graphite waste and found that mixed surfactants (LS-SDS) provided better milling efficiency as well as better control of slurry viscosity build-up during ultrafine graphite production. An anionic surfactant (SDS) provides electrostatic stabilization for the ground slurry. An anionic polymeric surfactant (LS) provides strong steric repulsion from a high-molecular-weight polymeric dispersant and electrostatic repulsion. Na^+^ from LS and SDS also provided strong ionic repulsion, increasing the distance between milled particles and improving the dispersion and viscosity of milled graphite slurry.^49^ Using this combination of SDS and LS, these two stabilization mechanisms were expected to help reduce the yield stress of ground slurry and help control the build-up of viscosity during production.^50,51^

### Comparison of Industrial Ball Milling and Bead Milling Processes on Milling Performance

The ball milling process has generally been used in commercial particle production. However, this technique is unsuitable for commercial production due to the prolonged milling time. Modern milling techniques, such as bead milling, were introduced with the expectation that they could shorten the production time, while their limitation was the solid loading in the operation. In this work, the milling performance, including size reduction and slurry viscosity over time for each process, was investigated.

**Figure 1:**
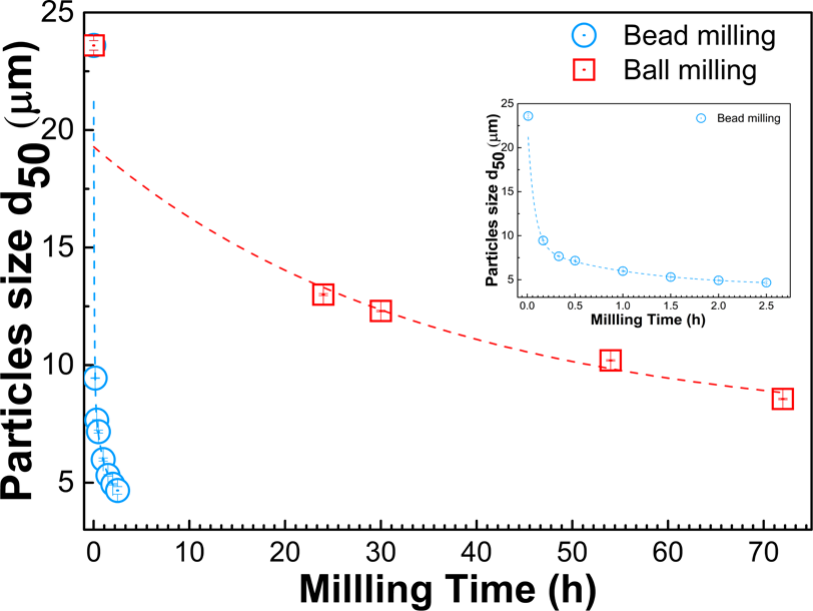
Particle size *d*_50_ reduction during 0–72 h of ball milling and 0–2.5 h of the bead milling process.

Bead milling was performed for 2.5 h with regular sampling during milling. Ball milling was carried out for 72 h. Slurry samples were collected during the milling operation. The particle size reduction over milling time between bead milling and ball milling processes is presented in Figure 1. In the ball milling process, the particle size of graphite scrap decreased gradually over the milling time in the first 24 h of the process. The particle size was reduced from the initial particle size *d*_50_ of 23.6 to 13 μm, but the particle size seemed to reduce at a lower rate afterward. The viscosity built up during milling, which resulted in insufficient milling performance.^27,28^ The particle size reached an ultrafine range at 72 h of ball milling with a particle size of approximately *d*_50_ = 8.56 μm. Compared to the ball milling process, the bead milling technique could produce ultrafine graphite scrap with a dramatically shorter process time. The average particle size *d*_50_ dropped to the ultrafine range of 9.45 μm within the first 10 min of the milling. The bead milling process was able to reduce the graphite scrap particle size to smaller than 5 μm after 1 h of milling, as shown in Figure 1. At this point, however, the particle size reduction rate was slower than the first 30 min of milling. This result indicated that bead milling could also be used for finer particle production for an application that requires smaller graphite than in the ultrafine range.

**Figure 2:**
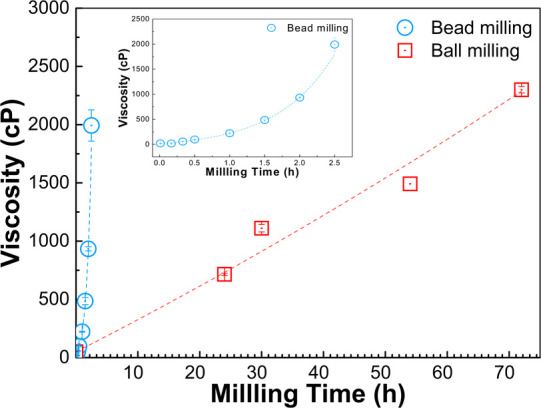
Slurry viscosity over milling time during 0–72 h of ball milling and 0–2.5 h of bead milling.

Slurry viscosity is an important parameter in the milling process. Viscosity build-up occurred during the milling process and decreased the milling efficiency. To avoid the viscosity build-up, chemical additives such as surfactants have been used for lowering the viscosity during milling. In this experiment, a sufficient amount of surfactants was used to control viscosity.^40,52,53^ The slurry viscosity process from both the bead and ball milling processes, as shown in Figure 2, revealed an increase in the viscosity as the milling time increased. The results revealed an exponentially improving rate of viscosity in bead milling and a linear increase in the ball milling process. Bead milling had better slurry viscosity build-up over time, as seen in Figure 2. Viscosity build-up exhibited in both techniques was caused by the reduction of particle size and the increase in the surface area, augmenting the liquid–solid and solid–solid interface of the slurry.^54,55^ The highly viscous slurry could also affect the milling operation, especially the bead milling process. This study found blockage inside the bead milling machine after milling for more than 2.5 h. The maximum operating viscosity range for the bead milling process was also determined in this experiment. The maximum slurry viscosity at which the bead milling could operate before the blockage was 2000 cP. For bead milling, the slurry viscosity should be kept lower than 2000 cP to avoid the slurry flow problem.

This investigation presented better performance when using the industrial bead milling technique with a high solid loading of 23 wt %, with acceptable slurry viscosity within an operable range. To utilize the same amount of graphite as ball milling (200 kg), the bead milling provides 9 times faster production than the ball milling process (approximately 8.17 h for 200 kg of ultrafine graphite scrap, with the premixing time included). To confirm the potential of using a bead mill to substitute a ball mill, however, the particle characteristics of milled ultrafine graphite scrap particles from both techniques were characterized before further use as a carbon filler in carbon block manufacturing.

### Effect of the Industrial Milling Process on Particle Characteristics

Particle breaking mechanisms in size reduction techniques could be separated into 2 main types, namely, impaction and shearing, depending on the grinding media movement.^56^ A different milling type could also provide different breakage mechanisms, which directly influence particle characteristics such as size distribution, particle morphology, and crystallinity.^57−59^ These particle properties are important parameters that affect the carbon block properties. Although the milling performance of bead milling yields better particle characteristics, a comparison should still be made to observe the detailed characteristics.

**Figure 3:**
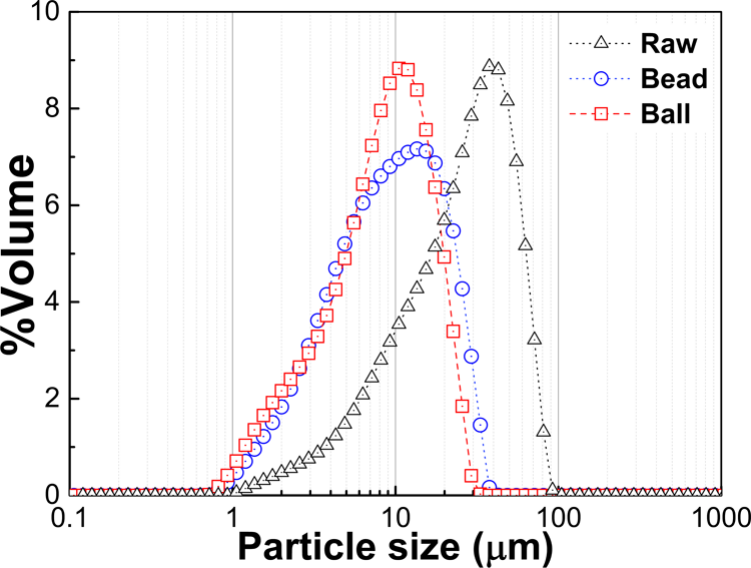
Particle size distribution of raw graphite scrap, 10 min bead-milled slurry, and 72 h ball-milled slurry.

**Figure 4:**
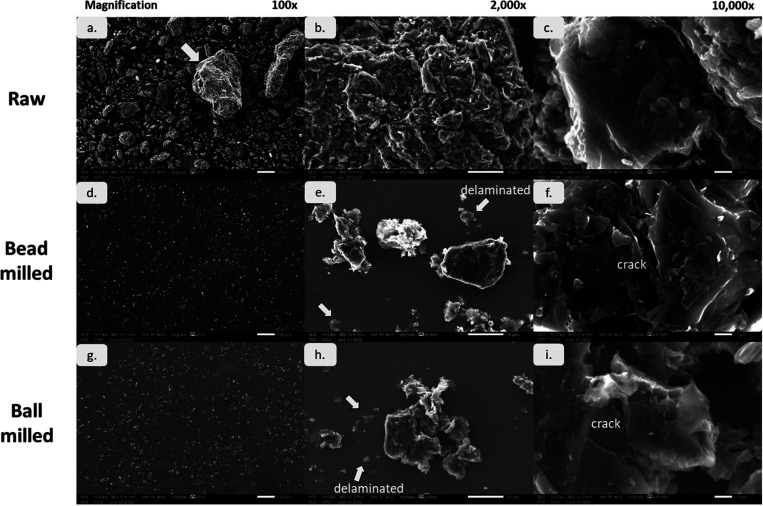
SEM images of raw graphite scrap (a–c) and bead-milled (d–f) and ball-milled (g–i) ultrafine graphite scrap at 100, 2000, and 10,000× magnification.

Raw graphite scrap collected from the cutting and tooling process of synthetic graphite blocks had a wide particle size distribution ranging from 1 to 100 μm, with most populations larger than 10 μm with a *d*_10_ of 5.91 μm, a *d*_50_ of 23.6 μm, and a *d*_90_ of 49.6 μm (Figure 3). Both the distribution and size parameters indicated the variation in raw graphite scrap particle size. SEM images of raw graphite scrap particles are shown in Figure 4. At low-magnification SEM (Figure 4a), the shapes of graphite scrap particles were irregular and varied in size. The particles had a size ranging from a few micrometers to hundreds of micrometers. The coarse particle size of the graphite scrap affected the carbon block properties. Large filler particles provided lower block mechanical properties and higher porosity.^19,60^

At a higher magnification (Figure 4b), the surface of a large particle, as indicated by arrows, revealed an agglomerate of smaller graphite particles as opposed to pristine graphite particles. This was because graphite scrap originally came from graphite blocks, which are a combination of graphite and a graphitized binder. At a 10,000× magnification (Figure 4c), the shape of the raw graphite scrap particle is flake-like and stacking, which is the nature of graphite.

The particle size distribution of milled graphite scrap obtained from ball-milled and bead-milled ultrafines is presented in Figure 3. Volume-based size parameters are listed in Table 1. Ball-milled graphite scrap size parameters were a *d*_10_ of 2.44 μm, a *d*_50_ of 8.56 μm, and a *d*_90_ of 18.1 μm. For bead-milled ultrafine graphite scrap, the size parameters were a *d*_10_ of 2.76 μm, a *d*_50_ of 9.12 μm, and a *d*_90_ of 22.3 μm. These results presented a slight difference in average particle size *d*_50_ between bead-milled and ball-milled graphite. The *d*_90_ of bead-milled graphite scrap was larger than that of ball-milled graphite scrap, correlating with the span of the distribution, which was 2.14 in bead-milled and 1.83 in ball-milled particles. The wider distribution of bead-milled ultrafine graphite scrap was due to the difference in the milling operation. The bead milling process ran at a high milling speed and pump rate, resulting in a fast transfer of slurry between the mixing tank and grinding chamber. As a result, the feed materials only spend a short period inside the milling chamber. By contrast, all of the material in the ball milling process stayed inside the milling chamber until the process was finished. All particles were ground continuously at the same time.

**Table 1:** Particle Volume-Based Size Parameters, Specific Surface Area, and Crystalline Parameters of Raw and Milled Ultrafine Graphite Scrap

	***d***_**10**_ (μm)	***d***_**50**_ (μm)	***d***_**90**_ (μm)	**SSA** (m^2^/kg)	***d***_**002**_ (Å)	***L***_**c**_ (nm)	***L***_**a**_ (nm)
raw	5.91	23.60	49.60	450	3.37	514	309
bead-milled	2.76	9.12	22.30	1001	3.37	179	315
ball-milled	2.44	8.56	18.10	1099	3.37	203	299

The specific surface areas (SSA) reported from Malvern in particle size distribution characterization of raw graphite scrap and bead- and ball-milled ultrafine graphite scrap were 450, 1001, and 1099 m^2^/kg, respectively. This result shows the increase in the surface area of the sample after being milled. After milling, the SSA increased by over 100% due to the size decrease from 24 to 9 μm. When comparing between bead-milled and ball-milled samples that were of similar size, however, it can be seen that there was only a minor difference between the SSA of milled samples, which indicates the similar surface area of the milled particles.

The particle morphology of raw graphite scrap and milled ultrafine graphite scrap was observed by SEM (Figure 4). The agglomerates of raw graphite scrap were drastically reduced into ultrafine size after milling with ball and bead milling while maintaining a flake-like shape. At a high magnification (10,000×), as in Figure 4f,i, cracking appearing on the particle surface produced from both techniques was observed, indicating the lateral breakage or fracture of the particle.

From Figure 4e,h, the thinner layer of graphite was also found in both ball milling and bead milling processes due to the delamination of the graphite stack caused by the shearing of grinding media.^31,56^ The particle morphology of both ball-milled and bead-milled ultrafine graphite shows irregular shapes of graphite particles with the presence of stacking and delaminated particles. Similar results were reported^31^ where the products from IsaMill and conventional ball mills showed similar particle characteristics.

**Figure 5:**
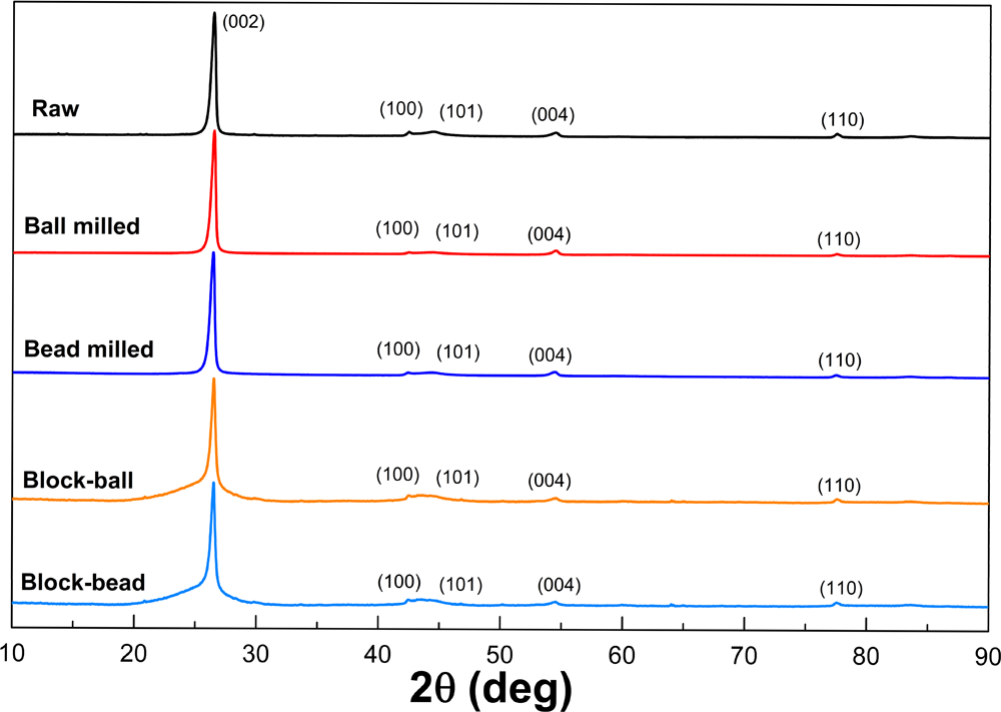
XRD spectra of raw graphite scrap and ultrafine graphite scrap from ball and bead milling and a carbon block fabricated from milled ultrafine graphite scrap.

The crystallinity of graphite scrap before and after milling was characterized by XRD (Figure 5). XRD spectra show peaks at 26, 42, 44, 54, and 77° 2θ, which correspond to the (002), (100), (101), (004), and (110), respectively. Crystal parameters were calculated using eqs 1–3 and are summarized in Table 1, where *d*_002_ was evaluated from (002) and *L*_a_ and *L*_c_ were calculated from (002) and (110). The interlayer spacing *d*_002_ was 3.37 Å for raw graphite scrap and 3.37 and 3.37 Å for bead-milled and ball-milled ultrafine graphite scrap, respectively. The stacking height (*L*_c_) of ultrafine graphite scrap after milling was reduced in both ball- and bead-milled samples, which resulted from the delamination of the graphite stack, corresponding to the delamination observed in SEM images. The small difference between the two samples was also found in the crystalline size (*L*_a_). The crystalline size was 315 and 299 nm for bead-milled and ball-milled ultrafine graphite scrap, respectively. The milled graphite scrap from both ball milling and bead milling processes exhibited the characteristic peak of graphite; when comparing the crystalline parameters, only a minor difference was found.

These particle characteristics substantiate a similarity of the output particle from ball-milled and bead-milled processes, where they exhibit similarities in the particle morphology breaking mechanism and crystallinity, despite differences in the milling parameters and energy. Finally, ultrafine graphite scrap powder from both ball-milled and bead-milled materials will be fabricated into carbon blocks with the same procedure to further compare the qualities of the final product.

### Carbon Block Product Properties

To explore the potential of the bead milling process to substitute conventional ball milling for ultrafine graphite scrap production for carbon block industries. The ultrafine graphite scrap powder from bead milling and ball milling was fabricated into carbon blocks. The microstructure and block properties, such as density, flexural strength, hardness, and resistivity, were evaluated.

**Figure 6:**
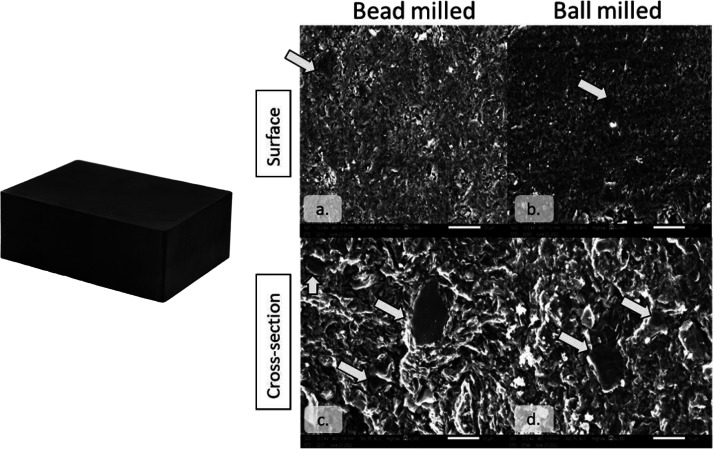
Carbon block and microstructure on the surface (a,b) and cross section (c,d).

The carbon block was cut into smaller specimens, and the surface of specimens was polished using no. 1200 sandpaper. The surface and cross section of carbon blocks fabricated from ball-milled and bead-milled graphite scraps were examined using SEM (Figure 6). Images revealed that the surface of the carbon block, both ball- and bead-milled samples, had pores on the surface. The presence of graphite particles in the block microstructure was also shown in the SEM image, indicated by arrows on both the surface and cross section of the carbon block. Discerning a significant difference between the surface characteristics of the ball-milled and bead-milled carbon blocks was challenging. The carbon block from bead-milled ultrafine graphite scrap appeared to have the same microstructure as the carbon block from ball-milled ultrafine graphite scrap. To verify the similarities of carbon blocks fabricated from ball-milled and bead-milled ultrafine graphite scrap, the mechanical and electrical characteristics of carbon blocks were examined.

From the XRD spectrum of the carbon block (Figure 5), the carbon blocks fabricated from bead-milled and ball-milled ultrafine graphite exhibited the same characteristic peaks with the same shape and the same *d*_002_ of 3.36 Å. If comparing the spectra of the carbon block to the XRD spectra of milled graphite powder, changes in crystallinity in the plane (002) were observed. Increasing the amorphous structure after fabrication into the carbon block was observed by the wider (002) peak and a decrease of *d*_002_ for the carbon block compared to milled graphite powder. This is due to the origin of the block, which is formed by the mixture of graphite, amorphous carbon, and coal tar pitch, resulting in an amorphous characteristic in the plane (002) of the carbon block.

**Figure 7:**
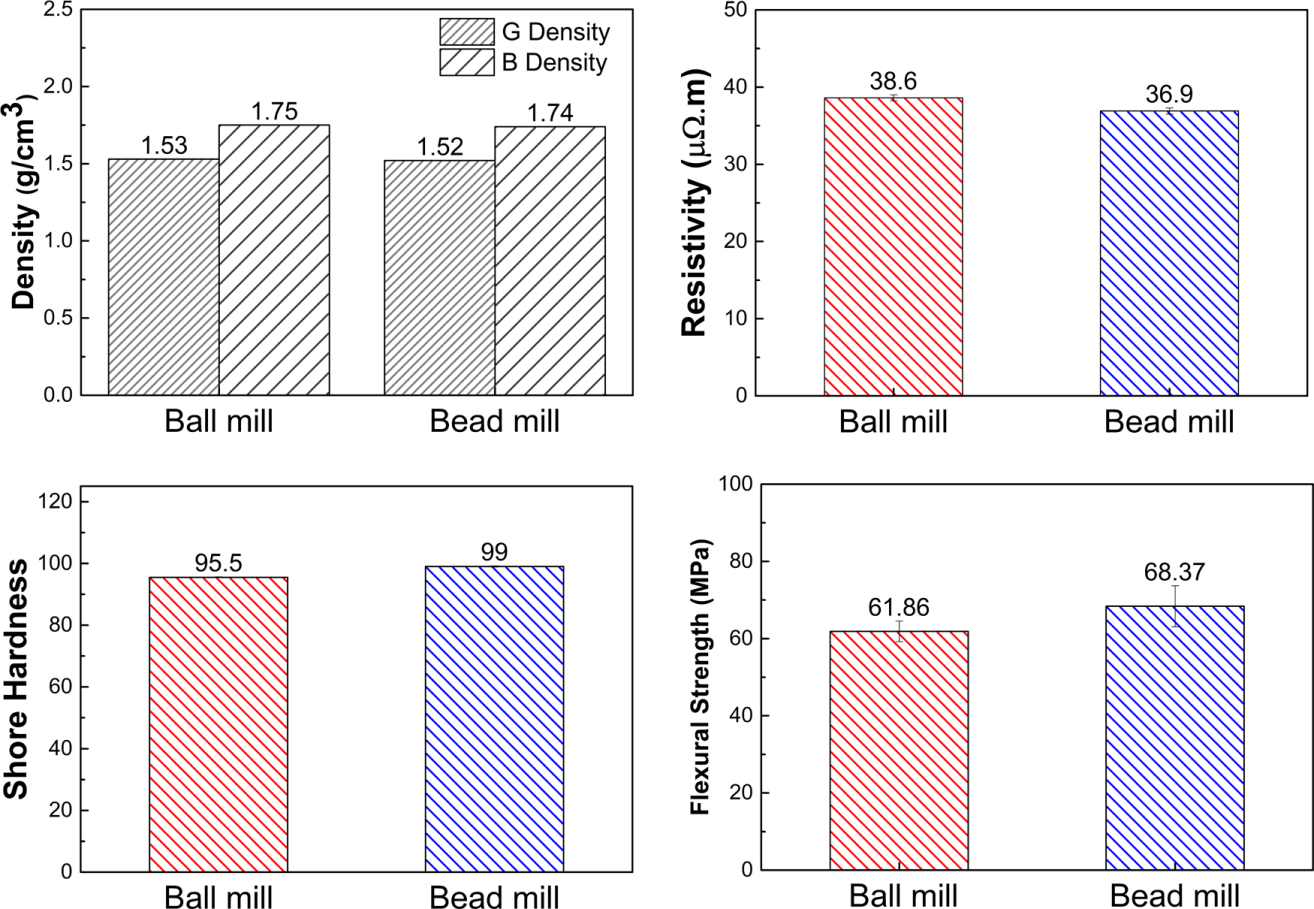
Density, shore hardness, flexural strength, and resistivity properties of ball-milled carbon blocks and bead-milled carbon blocks.

The mechanical and electrical properties of the fabricated carbon blocks are shown in Figure 7. The density of the carbon blocks, before and after heat treatment, was comparable between the two samples, with values of 1.53 and 1.75 g/cm^3^ for the ball-milled block and 1.52 and 1.74 g/cm^3^ for the bead-milled block, respectively. The resistivities of the carbon blocks were 38.6 and 36.9 μΩ·m for the ball- and bead-milled blocks, respectively. Additionally, the hardness of the bead-milled carbon blocks was found to be slightly higher than that of the ball-milled carbon blocks, at 99.00 and 95.50, respectively. Similarly, the flexural strength of the bead-milled carbon block was slightly higher than that of the ball-milled carbon block at 68.37 and 61.86 MPa, respectively. These results suggested that the properties of the carbon blocks produced from ball- and bead-milled ultrafine graphite scrap were comparable and, in some cases, identical, despite the minor differences in the crystallinity of the particles. This implies that the change in the size reduction process from ball milling to bead milling had no significant effect on the properties of the carbon block. Further, the properties of these carbon blocks were not different from typical carbon blocks.

The properties of carbon blocks fabricated from ultrafine graphite scrap and produced by two processes, namely, ball milling and bead milling, were examined. The results demonstrated that the microstructure, electrical properties, and mechanical properties of the carbon blocks, using milled ultrafine graphite filler from both ball and bead milling processes, were similar and provided the same quality. This outcome could substantiate the potential of bead milling as a substitute for ball milling in the production of ultrafine graphite scrap for the carbon block industry.

## Conclusions

The ultrafine graphite scrap was produced by 72 h of ball milling and 10 min of bead milling with particle sizes *d*_50_ of 8.56 and 9.45 μm, respectively. According to the SEM images, the morphology of the output particles from bead and ball milling was similar with a minor difference in the particle crystalline parameters. Milled ultrafine graphite had the same *d*_002_ of 3.37Å in both samples. *L*_c_ was 179 and 203 nm in bead- and ball-milled graphite, and *L*_a_ was 315 and 299 nm, respectively. Despite the difference in particle crystallinity, the properties of the carbon block product fabricated from bead-milled ultrafine graphite had comparable mechanical and electrical properties to those of the block from ball-milled ultrafine graphite. The carbon blocks from both processes showed identical microstructures and a slight difference in block density before and after heat treatment at 1.52 and 1.74 g/cm^3^ for the block from bead milling and 1.53 and 1.75 g/cm^3^ for the ball-milled block. Block hardnesses were 99.00 and 95.50 for bead- and ball-milled blocks, respectively. Similarly, the flexural strength of the bead-milled carbon block was 68.37 MPa, slightly higher than 61.86 MPa for the ball-milled block. The resistivity of the bead-milled and ball-milled carbon block was 38.6 and 36.9 μΩ·m, respectively. These results showed that bead milling could be used in high-solid-loading production (23 wt % graphite), and changing the milling process to bead milling could reduce the production time while maintaining the same particle output and carbon block product quality.
